# Gene Expression Analysis for Drought Tolerance in Early Stage of Potato Plant Development

**DOI:** 10.3390/biology13110857

**Published:** 2024-10-23

**Authors:** Rakhim Kanat, Malika Shamekova, Zagipa Sapakhova, Maxat Toishimanov, Dias Daurov, Nurgul Raissova, Zhanar Abilda, Ainash Daurova, Kabyl Zhambakin

**Affiliations:** 1 Institute of Plant Biology and Biotechnology, Almaty 050040, Kazakhstan; 2 Tanir Research Laboratory, Almaty 050060, Kazakhstan

**Keywords:** *Solanum tuberosum* L., potato, drought, abiotic stress, gene expression, selection

## Abstract

Drought has increasingly affected the yield of *Solanum tuberosum* L. (potato) every year over the last decade, posing serious economic problems for the global agricultural industry. Therefore, it is important to research drought tolerance in plants and obtain more robust varieties of crops. The aim of the present work was to study the expression of drought-upregulated genes in drought-tolerant and drought-sensitive varieties of potato. Bioreactors were used to identify whether each variety was drought-tolerant or drought-sensitive; then, expression analysis was performed according to the morphological characteristics of the plantlets in two different media: Murashige and Skoog (MS) medium and MS medium with 20% PEG-6000 to simulate osmotic stress. Based on the quantitative parameters of six initial varieties, two varieties were selected (Gala and Aksor) for further gene expression analysis. The expression of genes commonly upregulated in drought (*ER24*, *TAS14*, *DREB147315*, *PP2C*, *102605413* and *NF-YC4*) was higher in the drought-tolerant variety than in the sensitive one. Therefore, the expression of these genes can be used to determine the drought tolerance of a potato variety in vitro in the early plant development stage. Moreover, comparative analysis showed that some of the targeted genes used to identify drought tolerance in this study are conserved across different plant species.

## Simple Summary

Every year drought causes losses in yield of many crops including potato and causes significant economic problems. One of the ways to overcome drought issues is developing varieties that can withstand water scarcity better. In this study, we simulated osmotic stress using PEG-6000 and compared morphological characteristics of different varieties to identify contrasting varieties in drought tolerance in the early plant development stage. As a result, two contrasting varieties were analysed for gene expression, where clear differences in gene expression profiles were shown. These experiments allow to identify drought tolerance properties of variety in the early development stage of potato. Furthermore, comparative analysis showed that expression profiles of subset of drought-tolerance genes used in this study were also conserved across different crops.

## 1. Introduction

Drought years have become more frequent in the last decade [1], causing significant losses in crop yield, some estimating up to 35% for moderately drought-tolerant and up to 16% for drought-tolerant varieties [2]. Drought significantly increases the costs of agricultural production. Research on plants’ tolerance to drought is becoming increasingly important to minimize the impact of global climate change on agriculture [3]. Most crops are grown in water-deficient regions of the world [4]. In particular, in Kazakhstan, potatoes are grown in drought conditions in the southern foothill regions and over significant flat areas in the north of the country. Breeding potato plants for drought tolerance is important because climate change will increase the frequency of drought.

Conventional methods of breeding potatoes for drought tolerance are very labor-intensive, season-dependent, time-consuming and costly [5]. Vegetation indices, such as the leaf area index and aboveground biomass, have been found to be the main determinants of yield in tested varieties under drought conditions [6]. It should also be considered that early drought reduces the number of tubers per plant across all genotypes, and later drought starting from the moment of tuber initiation differs from variety to variety and is more pronounced in early varieties [7]. In addition, the ability of plants to recover after the elimination of soil drought should be considered, as this is a good indicator of their sensitivity to soil drought and allows the prediction of potato tuber yield [8]. For breeding programs, it is useful to identify physiological traits in potato varieties that are closely related but that differ in their tolerance to drought. However, evaluation based on morphological traits alone is often not effective. Therefore, various modern biotechnological methods are often used in breeding practices worldwide, with success.

In particular, in vitro cultivation methods are widely used to breed crops for tolerance to abiotic and biotic stresses. As a rule, drought stress conditions are modeled using polyethylene glycol (PEG) [9,10,11], including for potato [12,13]. Using PEG in in vitro culture to breed for tolerance to stress factors in the growing environment is efficient, as this approach can be used all year round, regardless of the season [14]. In the breeding process for drought tolerance, a high correlation between the results of in vitro experiments and field trials on soybean [15], rice [16], as well as for the plant growth and tuber yield of potato, has been shown [17].

In addition, the use of molecular markers has greatly facilitated the breeding process of almost all crops. At the same time, the number of molecular markers associated with certain breeding-valuable traits of agricultural crops is rapidly increasing. In particular, allelic differences between potato varieties were identified for five SSR markers, which showed a significant association with drought sensitivity. In all cases, an additional allele was predominant in the group of drought-sensitive varieties, indicating that selection against these alleles by marker-assisted crossing may confer drought tolerance to the hybrid [18]. Putative drought tolerance genes were differentially expressed in leaves of genotypes under water stress. The induction of *protein phosphatase 2C* gene was positively associated with yield maintenance under drought conditions. In addition, tolerant varieties expressed the transcription factor DREB to a greater extent than more sensitive varieties [6].

Drought stress causes changes in the expression of a number of genes that control morphological, physiological and biochemical traits at the level of transcription [19]. Transcriptomic changes in plants during drought stress provide insight into the mechanisms by which plants stabilize their metabolic processes to cope with drought conditions. In potatoes, understanding such drought-induced transcriptomic changes is crucial because prolonged field drought interferes with tuber formation and the developmental period of potatoes, which ultimately affects yield [20]. At the same time, it has been determined that drought stress turns on genes that are involved in the biosynthesis of endogenous hormones, which in turn, affect the expression of downstream genes associated with heat shock proteins [21]. Next-generation sequencing and single-molecule real-time sequencing technologies were used to study potato transcription profiles in response to drought stress modeling (20% PEG). This resulted in a significant improvement in the annotation of drought-induced genes in potatoes and provided a transcriptomic insight into the molecular basis of potato drought tolerance [22]. It was determined that in tomato, overexpression of the *TAS14* gene under drought conditions leads to tolerance to osmotic stress by reducing osmotic potential and accumulation of solute substances such as sugars and K+. Moreover, overexpression of the *TAS14* gene increased Na+ accumulation only in adult leaves, whereas in young leaves the accumulated solutes were K+ and sugars, suggesting that plants with overexpression of the *TAS14* gene are able to distribute Na+ accumulation between young and adult leaves over a long period under stress conditions [23]. Simultaneously, transcriptome analysis of genes associated with photosynthesis determined that different genotypes can respond differently to drought at the leaf level, but lose tuber productivity to the same extent [24].

It is advisable to conduct the most extensive studies of enzymatic activity in order to have a complete picture of the functional consequences observed in modeling drought in potatoes. The regulation of drought tolerance related genes (*DRO, ERECTA, ERF, DREB* and *StMYB*) has been shown to differ between potato varieties with high and low-stress tolerance index [17]. Studies also showed that EF1α and sec3 were the most stably expressed genes in potatoes under drought and osmotic stress [25]. It was determined that genes related to proline degradation were inhibited by PEG-induced stress in in vitro seedling culture, which leads to proline accumulation. In this case, drought-tolerant potato genotypes accumulate less proline than drought-sensitive genotypes, and proline accumulation is inversely correlated with root length [26]. It was shown that drought stress was reflected not only in drought-responsive genes associated with altered cell structure and components, but also in enhanced gene expression or “memory” of drought-responsive genes [27].

During drought stress plants employ adaptive measures to withstand osmotic and oxidative stress by initiating signals that modify present metabolism to restore homeostasis. Ethylene is a phytohormone known for inducing plant response to various stresses including drought, heat shock, osmotic stress, oxidative stress, and wounding, and activates plant defense [28,29,30,31]. It is a gaseous plant hormone controlled by many factors including *ER24* with the molecular function of promoting the growth of root systems and sustaining metabolism, helping plants sustain the drought environment [32]. Another transcription factor expressed during drought is Nuclear factor Y (NF-Y), well regulated in plants during microbe attack, root development and different stresses [33,34,35].

Previously, researchers studied potato transcriptional responses in drought and osmotic stress conditions [36,37,38], some studies focused on comparing different cultivar gene expression profiles between them using aeroponic systems [39] and mannitol [40] to simulate osmotic stress conditions. Studies were conducted to study expression profiles of particular gene families of potatoes during drought and osmotic stress such as *CDPK* [41], *CIPK* [42], *WD40* [43], *DUF668* [44] comparing homologous genes between each other. Similar studies comparing gene expression profiles of contrasting varieties were conducted on other crops like *Saccharum officinarum* (sugarcane) [45]. The aim of this work was to determine whether the expression patterns of commonly upregulated genes differed between drought-tolerant and drought-sensitive varieties and whether there were common, conserved genes whose overexpression improved drought tolerance across plant species.

## 2. Materials and Methods

### 2.1. Plant Materials

The potato variety Gala and local Kazakh varieties Yagodnyi-19, Aksor, Tyanshanskyi and Shagalaly from the in vitro potato collections of the Institute of Plant Biology and Biotechnology were used in the experiment. The origins of these cultivars are provided in Table 1. A brief description of each variety can be found in Supplementary Materials S3.

### 2.2. Plant Propagation and Osmotic Stress Application

The 30 internodes were planted using Murashige and Skoog (MS) medium (4.43 g/L of an MS nutrient medium with vitamins (PhytoTechnology Lab, Lenexa, KS, USA), containing 0.8% agar (Santa Cruz Biotechnology, Dallas, TX, USA), 3% sucrose (Sigma-Aldrich Chemie GmbH, Steinheim, Germany)) with cefotaxime antibiotics (100 µg/L) in a bioreactor (RITA^®^ systems (Cirad, Vitropic, France)) one week before forming roots with an immersion frequency of 3 h for 10 min, and air pressure of 0.5 bars as optimized by another study [46]. The rooted plantlets were transferred to a liquid MS medium: control medium or medium with 20% PEG-6000. Each variety was grown for 14 days at 24 °C with a 16/8 photoperiod. During 14 days, the morphological traits of plantlets (plant height, number of internodes and number of leaves), roots’ attributes (length and number) and maximum quantum efficiency of PSII were recorded every day. The maximum quantum efficiency of photosystem (PS) II (Fv/Fm) of leaves was determined using a portable chlorophyll fluorometer (Hansatech Instruments Pocket PEA, Serial Number 1947, Norfolk, UK) after 30 min of dark adaptation in the second and third leaves (from the top) of each plantlet [47].

### 2.3. Selection of Drought-Tolerant and Drought-Sensitive Varieties

The mean values of the potato traits were related using principal component analysis (PCA). Pearson’s correlation coefficients were used to examine the relationships between variables. The values for each trait were compared between groups using analysis of variance (ANOVA). Student’s criterion test was performed to detect significantly different mean values (*p* ≤ 0.05) to select contrast varieties. Statistical analysis was performed using JMP 17 PRO (JMP Statistical Discovery LLC, Cary, NC, USA) and R (version 4.4.1). Data visualization was performed using ggplot2 (version 3.5.1) [48] from tidyverse (version 2.0.0) [49] package in R. Pairs plots were created using GGally (version 2.2.1) package and Duncan’s multiple range test for expression data was performed using agricolae (version 1.3-7).

### 2.4. Plant Material for Gene Expression Analysis

The drought-tolerant “Gala” and its contrasting variety “Aksor” were chosen for gene expression analysis using bioreactor (RITA^®^ systems). Two-week-old plantlets were transferred to bioreactors containing MS medium and MS medium with 20% PEG-6000 for osmotic stress. Three biological replicates were used for the control and treatment. The roots and stems were collected at time points of 0, 1, 2, 4, 6, 8, 10 and 15 days, immediately frozen in liquid nitrogen and kept at −70 °C in a freezer for further RNA extraction and analysis [50].

### 2.5. Gene Expression Analysis Using qRT-PCR

Total RNA was extracted from cells or snap-frozen tissues using TRIzol Reagent, according to the manufacturer’s protocol. Extracted total RNA was treated with DNAse I (Thermo Fischer Scientific, Waltham, MA, USA) to remove any leftover contaminating DNA. PCR amplifications were performed using a RevertAid First Strand cDNA Synthesis Kit (Thermo Scientific^TM^, K1621). Prior to amplification, the RNA quality and concentration were determined using a NanoDrop 2000 spectrophotometer (Thermo Fischer Scientific, USA) [51]. Gene expression analysis was performed with the PowerTrack^TM^ SYBR Green Master Mix (Thermo Fischer Scientific, Waltham, MA, USA) using oligo(dT)_18_ in 15 µL reaction tubes. The protocols outlined in the manufacturer’s guidelines were followed. Expression data were normalized to Perox, and Actin mRNA expression and fold changes were calculated using the $2^{-\Delta \Delta C_{t}}$ method. The primers are presented in Table 2.

The expression of the *ER24*, *TAS14*, *DREB147315*, *PP2C*, *102605413* and *NF-YC4* genes was measured using quantitative real-time PCR with the CFX Connect^TM^ Real-Time System (Bio-Rad, Hercules, CA, USA) and normalized to the expression of Actin and Perox. The cycles for PCR amplification were set in accordance with the PowerTrack^TM^ SYBRTM Green Master Mix manufacturer’s protocol. Among genes that were regulated by drought in potatoes, we focused on those belonging to key categories playing an important role in drought response modulation such as hormones, transcription factors and abiotic defense responses. The primers used for the experiment are provided in Table 2.

### 2.6. Searching Criteria for Comparative Analysis

To compare the obtained results with those from other studies on potato and other crops, namely, tomato and wheat, studies were identified using the PubMed search tool with the following keywords: [*Solanum tuberosum* L., *Solanum lycopersicum* L., *Triticum aestivum* L.], drought, RNA-Seq. Studies providing raw data, consisting of multiple organs and involving drought tolerance, were selected for comparative analysis. Raw data from selected studies were downloaded and extracted using the NCBI-provided SRA toolkit.

Studies researching crops under drought in different phases and in different organs were downloaded using the SRA toolkit. These studies were identified through a PubMed search with the following keywords: *Solanum tuberosum* L., potato, drought, RNA-Seq. The selection criteria were as follows: RNA-Seq analysis, consisting of multiple organs, provided raw data or gene count, and preferably consisting of drought-tolerant and intolerant varieties for comparison.

Raw data from studies have been downloaded using the NCBI-provided sra-toolkit (version 3.1.0) according to their accession number given by the original authors. Raw data were evaluated for quality using FastQC (version 0.12.1) [55] and adapter sequence and ends were trimmed using fastp (version 0.23.4) [56]. Clean data were obtained by removing low-quality reads (reads containing more than 10% unknown nucleotides (N) and reads containing greater than 50% low-quality (Q-value ≤ 20) bases). Cleaned reads were aligned to the reference genome using Hisat2 [57], transcript compilation was conducted using StringTie [58], differential expression was performed using Ballgown [59] if gene expression matrix was not provided. The following reference genomes were used for read alignment: potato (SolTub_3.0), and tomato (SL3.1) from the NCBI genome database using NCBI’s datasets tool.

## 3. Results and Discussion

### 3.1. Morphological Analysis

PEG-induced osmotic stress on plant morphological traits in vitro is often used for the preliminary screening of crop genotypes for identifying drought tolerance. In particular, it is noted that the length of the seedling and root are the most important traits by which one can find out the drought tolerance of the genotypes. Moreover, these traits are important for most crops, such as soybean (*Glycine max*) [15], sorghum (*Sorghum bicolor*) [60], rice (*Oryza sativa*) [61], rapeseed (*Brassica napus* L.) [62] and tomatoes (*Solanum lycopersicum* L.) [9]. In all these experiments, stress led to a decrease in the length of seedlings and roots in vitro. At the same time, it is known that early drought reduces the number of tubers per plant in all studied potato genotypes [7]. Therefore, it would be logical to assume that modeling drought at the early stages of plant development will allow us to distinguish drought-tolerant and drought-sensitive potato varieties. In this study, the growth of in vitro plantlets was determined based on plant height, root length, number of internodes, number of leaves, number of roots, and maximum quantum efficiency of PSII of potato varieties Yagodnyi-19, Gala, Aksor, Tyanshanskyi, and Shagalaly during 15 DAT (days after treatment). All parameters were decreased (Supplementary File) in all evaluated varieties, except the maximum quantum efficiency of PSII of Yagodnyi-19 (0.685) and Tyanshanskyi (0.741) compared to the control (0.656 and 0.690, respectively). The plant height (PH) decreased especially in varieties Shagalaly (3.8 cm) and Aksor (6.4 cm) in comparison to its controls. The length of roots (LR) and number of internodes (NI) were reduced in Aksor (2.6 cm and 6.9) and Tyanshanskyi (4.5 cm and 6.6). The number of leaves decreased in Yagodnyi-19 (7.9) and Aksor (8.0), while the number of roots decreased in Yagodnyi-19 (10.2) and Shagalaly (9.8 cm). The most affected genotype was Aksor according to decreased number of morphological characteristics; in contrast, Gala showed tolerance responses to PEG-induced water stress (Supplementary Tables S6 and S7).

#### 3.1.1. Principal Component Analysis

**Figure 1:**
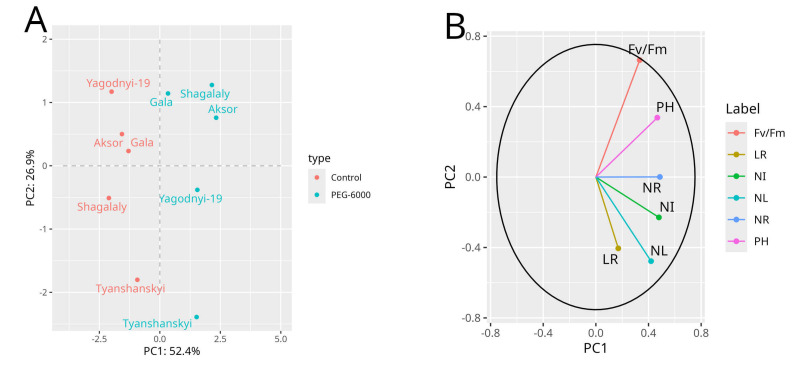
Principal component analysis (PCA) between main indicators of control and PEG-6000-treated plantlets. PEG-6000-treated and control plantlets form distinct clusters in two components. (**A**) PCA plot with two components (PC1: 52.8%, PC2: 25.9%), (**B**) Biplot maximum quantum efficiency of photosystem II (Fv/Fm), plant height in cm (PH), length of roots in cm (LR), number of internodes (NI), number of leaves (NL), number of roots (NR).

In this study, principal component analysis (PCA) was used as a multivariate statistical analysis for possible differences in potatoes using the dependent variables, morphological parameters, which served as the main component (Supplementary Table S2). As shown in the Figure 1, PCA explained 83.1% of the total variation; PC1 explained 55.6% and PC2 explained 27.5%. This figure also shows that the group with no treatment and PEG-6000-treated groups formed separate clusters, and each group could be distinguished. The results show that all the morphological parameters were formed in the control condition, which shows that stressors influenced PEG-6000 treatment. The range of association between the first component (PC1) and all the evaluated variables ranged from 11% to 52%, and all of them were in the same direction. The second component (PC2) had a positive direction for maximum quantum efficiency of PSII, plant height and root length (68%, 42% and 36%, respectively) and a negative direction for other variables (Supplementary Figures S5 and S6). PCA revealed trait influences between the control and PEG-6000-treated groups, which is in agreement with previous studies [63,64].

**Figure 2:**
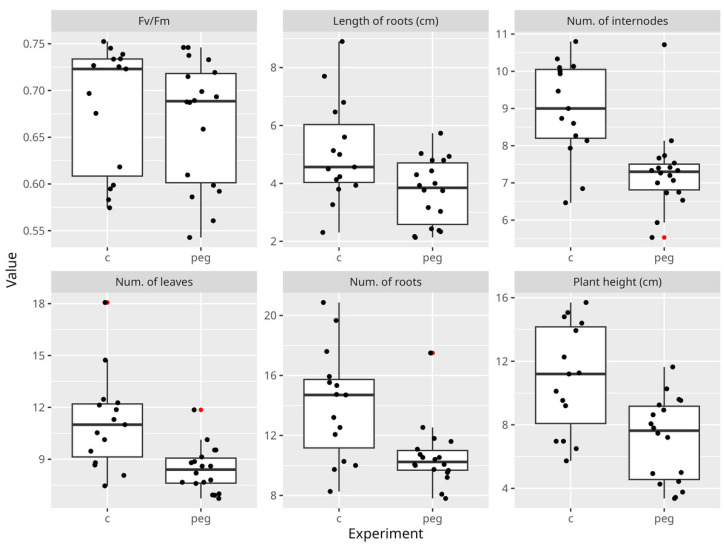
Boxplot showing difference between morphological characteristics of plantlets treated with polyethylene glycol (PEG)-6000 and control. Outliers shown as red dots.

Analyzing the reaction of potato plantlets treated with PEG-6000 in the nutrient medium, it was determined that there was variability in the studied traits in plants. At the same time, the number of leaves and root length changed but not dramatically (Figure 2), while the plant height, number of internodes and number of roots changed significantly (Figure 2).

Thus, all the studied traits can be used to characterize potato varieties for resistance to simulated osmotic stress at the level of in vitro plantlets. Many authors have noted the relationship between abiotic stresses and the work of photosynthetic apparatus [65,66,67]. Thus, the species *Triticum dicoccum* Schuebl. and *Triticum aethiopicum* Jakubz. are noted as having the most stable indicators of root system development and relatively high photosynthetic activity under conditions of modeling drought [68]. In addition, it was shown that stress-tolerant plants effectively improved growth and physiological and photosynthetic parameters in pistachio seedlings subjected to salinity and drought stress, which is explained by local adaptation under in vitro conditions [69]. Drought in potatoes is believed to significantly reduce the photosynthetic rate, transpiration rate, stomatal conductance and intercellular CO_2_ concentration in leaves, while stress reduces the net photosynthetic rate of potato plants [70].

#### 3.1.2. Correlation Analysis

**Figure 3:**
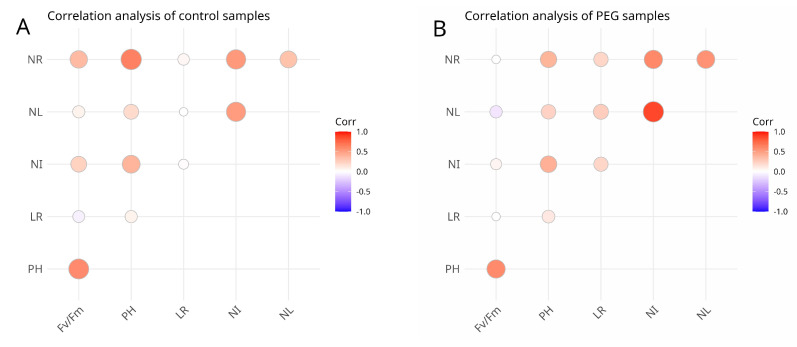
Pearson’s correlation analysis between morphological characteristics of control and PEG-6000-treated potato plantlets. Maximum quantum efficiency of photosystem II (Fv/Fm), plant height in cm (PH), length of roots in cm (LR), number of internodes (NI), number of leaves (NL), and number of roots (NR). (**A**) shows tcorrelation plot for control group, (**B**) shows correlation plot for PEG-6000 treated group.

**Figure 4:**
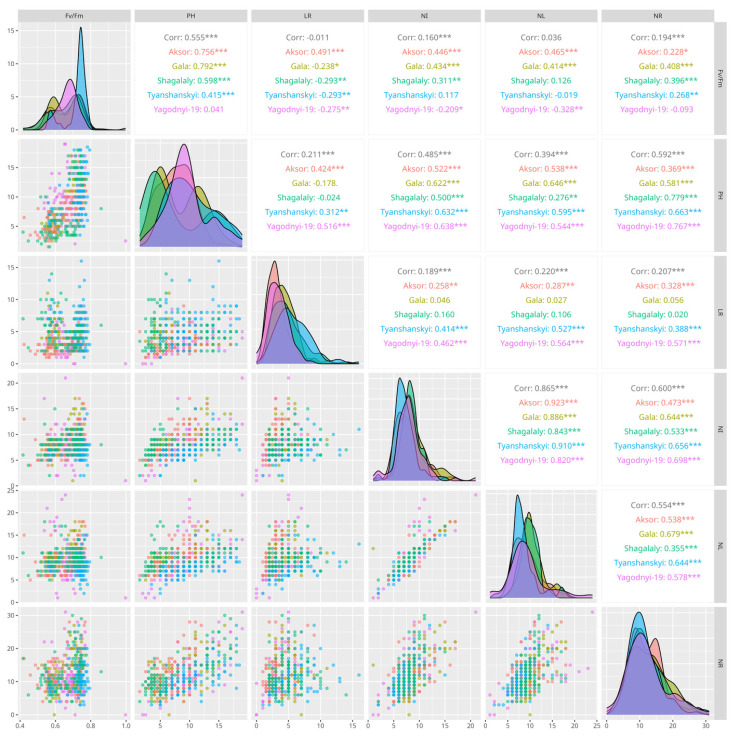
Pairs plot showing Pearson’s correlation coefficients between the morphological characteristics of the different potato varieties used in this study. A pairs plot showing separate correlations for the control and PEG-6000-treated groups can be found in the Supplementary Material. Maximum quantum efficiency of photosystem II (Fv/Fm), plant height in cm (PH), length of roots in cm (LR), number of internodes (NI), number of leaves (NL), and number of roots (NR). *p*-values of correlation tests are represented as follows: “***”—*p*-value is < 0.001; “**”—*p*-value is < 0.01; “*”—*p*-value is < 0.05; “ ”—otherwise.

Figure 3 shows the Pearson correlation coefficients between the quantitative traits in the control group. These correlations showed significantly positive correlations between plant height and root length (r = 0.7725), and the number of internodes and number of leaves (r = 0.8085). Figure 4 shows a correlation between quantitative variables combing both control and PEG-6000 treated groups with each variety separated (Supplementary Table S1). Although root length is related to drought tolerance, the authors of the study examining the relationship between root length and drought tolerance suggest that correlations are too weak and effect sizes too small to explain the differences in drought tolerance observed among potato varieties [71]. The maximum quantum efficiency of PSII had significant negative correlations with the number of leaves (r = −0.9732). Root length had a significant negative correlation with the number of internodes (r = −0.9013). The maximum quantum efficiency of PSII had no positive correlation between all parameters except root length (r = 0.3516) (Supplementary Figure S1). It is known that various stresses reduce leaf area, stem area and plant height [72], respectively, reducing the photosynthetic activity of potato [73].

Pearson correlation coefficients between the main parameters in PEG-6000-treated plantlets showed significantly positive correlations between the plant height and maximum quantum efficiency of PSII (r = 0.8558) and the number of internodes and number of leaves (r = 0.8054), as shown in the table (Supplementary Material). The maximum quantum efficiency of PSII showed significant negative correlations with the number of leaves (r = −0.9732). In correlation analysis, no significant negative correlations were found for the PEG-6000-treated group, but the number of leaves showed negative correlations with maximum quantum efficiency of PSII (r = −0.7704) and plant height (r = −0.6589). The results also showed that the maximum quantum efficiency of PSII had no positive correlation with any parameter except plant height (r = 0.8558). According to the correlation data obtained, it should be noted that the maximum quantum efficiency of PSII was generally negatively correlated with all the parameters in both the control and PEG-6000-treated groups, except for root length in the control and plant height in the PEG-6000-treated group, while in the control group, the maximum quantum efficiency of PSII, plant height and root length were negatively correlated with the number of internodes, number of leaves and number of roots. In the PEG-6000-treated group, only root length showed a weak positive correlation with the maximum quantum efficiency of PSII.The PEG-6000-treated group clearly showed higher correlations for some varieties (Yagodnyi-19: 0.708), while a very small change in correlation between the control and PEG-6000-treated groups was observed in some cases (Gala: −0.149; Shagalaly: −0.061) (Supplementary Figures S2–S4).

In our case, in vitro drought modeling also affected the maximum quantum efficiency of PSII, and the different genotypes studied responded differently to the stress in this regard. All the varieties showed greater upward and downward variability in the maximum quantum efficiency of PSII under stress than the control variant (Figure 2). Since all the investigated traits showed reliable variability in in vitro drought modeling with PEG-6000, we had an opportunity to distinguish the studied genotypes in terms of their sensitivity to drought conditions at the early development stage of the plantlets.

### 3.2. Selection of Drought-Tolerant and Drought-Sensitive Varieties

The presence of PEG-6000 in the nutrient medium affected almost all the potato varieties involved in the experiment (Table 3). Firstly, all the measured quantitative traits decreased. At the same time, the maximum quantum efficiency of PSII changed to varying degrees in different varieties. Thus, in the varieties Yagodnyi-19 and Tyanshanskyi, it increased in the presence of PEG-6000, and in other varieties, it decreased. The results showed that in the Gala variety, the number of internodes, length of roots and their quantity did not change significantly in PEG-6000-induced drought, while in the Aksor and Tyanshanskyi varieties, all the measured quantitative parameters decreased significantly in the presence of PEG-6000 compared to the control (Supplementary Table S3).

Plantlets of six potato varieties were grown in bioreactors with 20% PEG-6000 and bioreactors to compare the morphological properties between them and determine which varieties were tolerant and sensitive to osmotic stress. Prior plantlets were grown in vitro until root formation. Based on the results obtained (Table 3), Aksor was selected as the most sensitive to osmotic stress and Gala as a tolerant to osmotic stress variety to further study the expression of major genes expressed during drought to assess the difference in expression of these genes.Aksor showed a highly noticeable difference in the control and PEG-6000-treated groups in morphological properties compared to Gala, for which no significant difference was observed in the length of roots, number of internodes or number of roots. In conclusion, the variety Gala is the most tolerant among the studied varieties in terms of osmotic stress, while the Aksor variety can be considered the least tolerant.

### 3.3. Gene Expression Analysis

**Figure 5:**
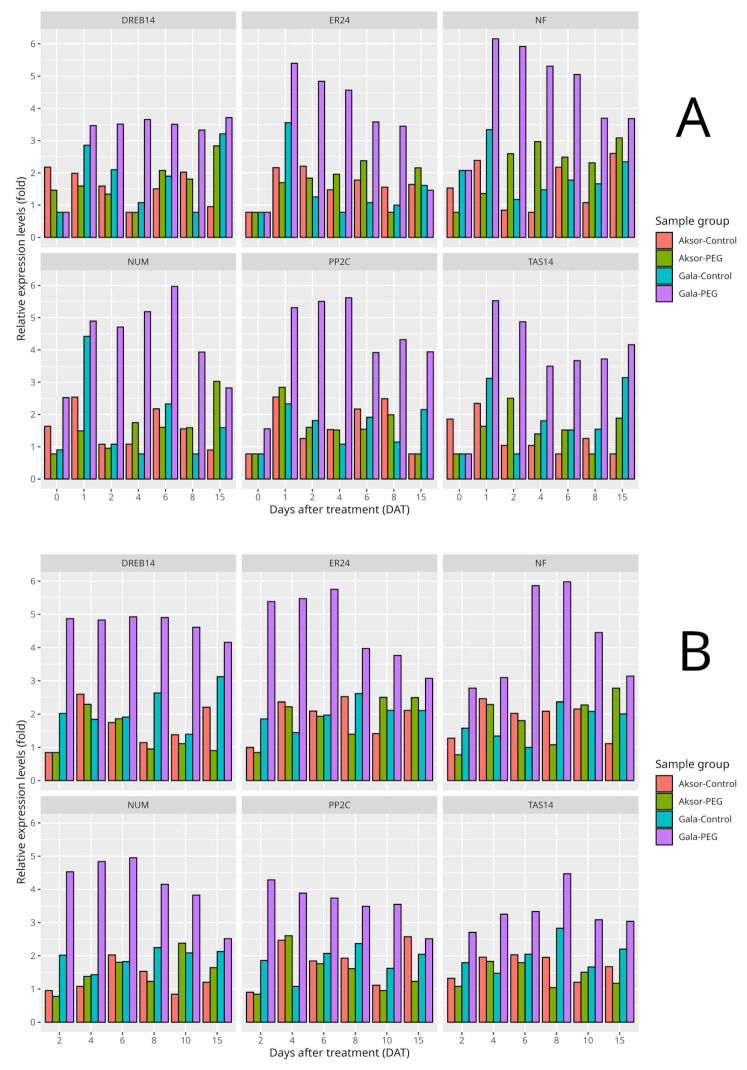
Relative expression levels of *DREB147315*, *ER24*, *NF-YC4*, *102605413*, *PP2C* and *TAS14* genes in Aksor and Gala varieties in two different MS media, with the other medium containing PEG-6000 to simulate osmotic stress. (**A**) Stem samples (**B**) Root samples.

The quantitative real-time PCR was performed under two conditions (control and PEG-6000) to determine the drought tolerance difference at the molecular level between the two varieties (Gala and Aksor). Figure 5 shows the expression of the genes *DREB147315*, *ER24*, *NF-YC4*, *102605413*, *PP2C* and *TAS14* in the roots and stems of control and PEG-6000-treated plantlets of the two varieties with contrasting osmotic stress tolerance properties. Results for stem samples at 0, 1, 2, 4, 6, 8 and 15 days after treatment (DAT) and for roots at 2, 4, 6, 8, 10 and 15 DAT are shown. Overall, all six selected upregulated genes showed a clear difference between the two varieties, the osmotic stress-tolerant Gala and osmotic stress-sensitive Aksor, in both the roots and stems across all days.The expression of DREB147315 in stem samples reached a high level in Gala at 1 DAT (with a relative expression level of 3.46) and showed little deviation across all days, while it increased and decreased a small amount in Aksor. This pattern was also noticeable in root samples. The overall expression of DREB147315 was higher in root samples compared to stem samples. *ER24* in both stems and roots showed high expression right after PEG-6000 treatment, declined slowly after 2 DAT in Gala (1 DAT: 5.4; 2 DAT: 4.83, 4 DAT: 4.56, 5 DAT: 3.58, etc.) and had low expression across all days in Aksor. *NF-YC4* gene expression was high one day after treatment with PEG-6000 in Gala, and started slowly decreasing in stems, slowly increased until 8 DAT and showed a further decline in root samples, while both the roots and stems of Aksor showed inconsistent and low NF expression. The *PP2C* gene showed overall high and similar expression across all days in Gala, while Aksor showed inconsistent and low expression. The expression of the *102605413* gene in the stems of Gala increased up to day 6 and, on 15 DAT, had a similar expression level to that on the first day. *TAS14*’s expression in stem samples of the Gala variety was the highest on 1 DAT, started slowly decreasing until 4 DAT and afterward showed consistent expression. No genes exhibited a noticeable change in the Aksor variety control and PEG-6000-treated samples (Supplementary Tables S4 and S5).

The sixth DAT showed the most significant change in the transcription of major genes in Gala. It can be seen from the figure that all the major drought-responsive genes are much more strongly expressed in the more osmotic stress-tolerant variety (Gala) than in the sensitive variety (Aksor). Leaf and root samples were selected and examined using targeted qRT-PCR for major genes regulated during drought (Supplementary Figures S8 and S9).

It was shown before that overexpression of the *DREB* genes in potato [74], wheat [75] and sugarcane [76] improves the drought tolerance of crops affected with a low amount of water. It is well known that genes involved in ethylene response (*ER24*) and *NF-YC4* genes were also upregulated in drought and other abiotic stresses in potato [77] and tomato [78,79]. The potato study also revealed the upregulation of the *TAS14* gene which was also selected for quantitative gene expression analysis. *PP2C* is a protein phosphatase gene, also commonly known to be upregulated during drought stress in potato [27,80] and other crops such as wheat [81]. Generally, presented genes are up-regulated in both varieties, but clearly in the drought-tolerant variety it had higher expression levels. This validates the claim that overexpression of genes up-regulated during droughts such as *DREB, TAS14* and others improves the drought tolerance of crops [23,82,83,84,85].

The major plant signal transduction pathway in abiotic stresses such as drought is the abscisic acid (ABA)-signaling pathway. Among many mechanisms controlling this pathway, phosphorylation and dephosphorylation, regulated by protein phosphatases are considered important signal transduction mechanisms [86,87]. Protein phosphatases type 2C (PP2Cs)counteract *SnRK2 kinases* by physical interaction, and thereby inhibit the activation of the transcription factors that mediate ABA-responsive gene expression. ABA modulation via negative feedback regulates the maintenance of plant homeostatis [88]. Stomatal closure control and respective reduction in the leaf area where water loss can occur are considered the main physiological mechanisms by which the ABA signaling pathway plays a role in plants during drought [89].

### 3.4. Comparison with Other Studies

Based on the criteria described in the Materials and Methods, three studies were selected involving potato [22], tomato [90] and wheat [91]. Using the criteria listed in the Materials and Methods, a number of studies were selected for comparative analysis. After aligning and annotating aligned reads, FPKM values were used for comparison. A *Solanum tuberosum* L. study involved potato seedlings as in the present study and applied drought stress. Using the raw data provided and methods described above, a table of expression counts was created for each gene that was aligned to the reference genome. Genes in the table (Supplementary Material) that were used in the current study were extracted, namely, *DREB147315*, *ER24*, *NF-YC4*, *102605413*, *PP2C* and *TAS14*. All six genes involved in the study were upregulated in drought studies of potato and tomato, which may be because they belong to the same group. Among the genes that were regulated by drought in seedlings, we focused on those belonging to key categories playing an important role in drought response modulation such as hormones, transcription factors and abiotic defense responses. In addition, the *DREB147315* gene, which is known as a major transcription factor involved in the responses to most abiotic stresses, was also found to be upregulated in all of the studies involving seedlings [92,93,94]. The most upregulated genes in this study were those responsible for the modulation of hormones, transcription factors and abiotic defense responses. In particular, *DREB147315, ER24, TAS14* and *PP2C* were upregulated in all the studies, including those on wheat.

Meta-analyses identifying key genes involved in drought across different species [95] and individual species such as wheat [96] and rice [97] have previously been published. This study used a similar approach, expanding the analysis across different crop species. The aim of this study was to investigate molecular markers of drought tolerance in potato varieties while also investigating markers conserved across crops instead of identifying specific responses in each crop. This study demonstrated that the drought tolerance of a variety can be identified using qRT-PCR on a small subset of genes responsible for drought stress. The study also shows that some genes upregulated during drought stress in potatoes can also be used for screening in other crop species such as wheat and tomato.

In the study [95], authors conducted a meta-analysis of transcriptome comparison across seven plant species, in particular, *Arabidopsis thaliana, Solanum lycopersicum* L., *Zea mays, Vitis vinifera, Malus domestica, Solanum tuberosum* L., *Triticum aestivum* L. including multiple organs and multiple developmental stages of plants.As a result of the comparative analysis, authors found 934 genes commonly regulated among three crops (*Solanum lycopersicum* L., *Malus domestica, Arabidopsis thaliana*) in seedlings and 132 genes commonly regulated among four species (*Solanum tuberosum* L., *Zea mays, Triticum aestivum* L., *Vitis vinifera*) in mature leaves.Also, the authors found that 22 genes are commonly regulated among both leaves and seedlings. This shows that there are molecular mechanisms against drought conserved across species and there is the possibility of choosing a small subset of genes to improve the time and efficiency of selection using molecular markers. For example, genes involved in ethylene signaling, DREB family genes and other abiotic stress response genes are commonly regulated in multiple crops.

The present work showed that, at least in the Solanaceae family, the chosen genes (*DREB147315*, *ER24*, *NF-YC4*, *102605413*, *PP2C* and *TAS14*) can be used to determine the drought tolerance of varieties and comparatively analyze multiple varieties using gene expression analysis. Expanding this gene set and selecting other sets with better performance are future possibilities. Also, combining gene expression analysis with other morphological analyses could improve the selection results. A previous study in wheat drought tolerance also showed that a higher stem dry weight and content of water-soluble carbohydrates correlated with drought tolerance [98].

## 4. Conclusions

The treatment with PEG-6000 in the nutrient medium affected almost all the potato varieties involved in the experiment. Most of the quantitative traits decreased, while the maximum quantum efficiency of PSII changed to varying degrees in different varieties. Gala was selected as the most osmotic stress-tolerant variety, showing no significant change in internode and root length between the control and PEG-6000-treated plantlets, while the Aksor variety showed significant decreases in all the quantitative parameters. Therefore, Aksor was selected as the sensitive variety and Gala as the tolerant to osmotic stress variety. Quantitative real-time PCR showed clear differences in drought tolerance gene expression regulation between Gala and Aksor in both root and stem samples, and almost all the genes upregulated during drought showed higher expression in Gala than in Aksor. The results obtained from the study show that the drought tolerance of potato varieties can be determined in the early stage of plant development through the gene expression analysis of genes commonly upregulated during drought (*DREB147315*, *TAS14*, *NF-YC4*, *ER24*, *PP2C* and *102605413*) as simulated with PEG-6000 medium. This study demonstrated that varieties with contrasting osmotic stress tolerance properties show vastly different expression patterns of the main drought-tolerance genes, which can be used for variety selection in the early stage. Comparative analyses were also carried out, showing similar levels of expression of drought-tolerance genes in different plant species, thereby validating claims that the overexpression of genes upregulated in drought improves the drought tolerance of varieties. In the future, morpho-physiological and molecular assessments of drought tolerance in contrasting (arid and non-arid) field conditions will be studied to confirm the hypotheses of the present studies.

## Figures and Tables

**Table 1:** *Solanum tuberosum* L. varieties used in this study.

Varieties	Origin
Gala	NORIKA GMBH, Germany
Yagodnyi-19	Northwest Agricultural Research Center
Aksor	“Kazakh Research Institute of Potato and Vegetables Growing”, LLP
Tyanshanskyi	“Kazakh Research Institute of Potato and Vegetables Growing”, LLP
Shagalaly	“Kazakh Research Institute of Potato and Vegetables Growing”, LLP × A.I. Barayev Research and Production Center for Grain Farming

**Table 2:** Primers used for the expression analysis of the six chosen genes.

Gene	Sequence	Reference
*Actin*	AGGAGCATCCTGTCCTCCTAA	[25]
	CACCATCACCAGAGTCCAACA	
*Perox*	AGCACTGATCCATCACATCCC	[52]
	TGGTAGTTGGAAAGATTGAGAAGC	
*ER24*	GAATCAGGCATTGCGAGCTGAATCAGGCATT	[52]
	GCCGCCTTCTTGTTCAATCGCCGCCTTCTTG	
*TAS14*	CAACAGCAGCTTCGTCGATCAACAGCAGCTT	[52]
	CATGTCCTCCTCCTGGCATCATGTCCTCCTC	
*DREB147315*	TGTTCATGGATGAGGAAGCG	[6]
	AACATTGGGGAGGAGGTAGCAT	
*PP2C*	TCACCGATTGCTCGAGACA	[53]
	GTCCCAATTCCTCTGTCCA	
*102605413*	AGTGAAGCATTTCGTAGAGCCA	[54]
	ACGATGAGTCATGGTTCTGCTT	
*NF-YC4*	CGGAGATACCCACCAACTCCGGAGATACCCA	[52]
	AAAGCTCGGTGGAACTAGCAAAGCTCGGTGG	

**Table 3:** Effect of the PEG-6000 in the nutrient medium during potato seedling growth. NS: not significant; the *p*-values are for comparisons between the control and PEG-6000-treated groups of each genotype. Maximum quantum efficiency of photosystem II (Fv/Fm), plant height in cm (PH), length of roots in cm (LR), number of internodes (NI), number of leaves (NL), and number of roots (NR).

Varieties	(Fv/Fm)		PH		LR		NI		NL		NR	
	**conc**	***p*** **-Value**	**conc**	***p*** **-Value**	**conc**	***p*** **-Value**	**conc**	***p*** **-Value**	**conc**	***p*** **-Value**	**conc**	***p*** **-Value**
Yagodnyi-19-C	0.656	0.0156	11.3	0.0001	3.8	0.0015	8.6	NS	13.4	0.0176	14.4	0.0005
Yagodnyi-19-PEG	0.685		8.2		2.9		7.5		7.9		10.2	
1-13 Gala-C	0.685	0.0005	9.7	0.0002	4.5	NS	9.5	NS	11.2	0.046	12.9	NS
Gala-PEG	0.635		7.0		4.3		8.5		10.1		12.3	
Aksor-C	0.686	0.0002	9.1	0.0001	4.1	0.0001	9.6	0.0001	11.1	0.0001	14.6	0.0001
Aksor-PEG	0.635		6.4		2.6		6.9		8.0		10.6	
Tyanshanskyi-C	0.690	0.0001	12.3	0.0001	7.8	0.0001	8.3	0.0003	9.4	0.0001	12.6	0.0275
Tyanshanskyi-PEG	0.741		9.1		4.5		6.6		7.3		10.3	
Shagalaly-C	0.690	0.0001	12.0	0.0001	5.2	NS	8.9	0.0001	10.6	NS	15.7	0.0001
Shagalaly-PEG	0.619		3.8		4.6		7.3		9.6		9.8	

## Data Availability

The raw data supporting the conclusions of this article are provided in Supplementary Materials.

## Supplementary Materials

The following supporting information can be downloaded at: https://www.mdpi.com/article/10.3390/biology13110857/s1, Table S1: Pearson’s correlation coefficients between morphological characteristics; Table S2: Eigenvector and variance explained associated with principal component; Table S3: Effect of the PEG-6000 in the nutrient medium; Table S4: relative expression level expression data of each sample; Table S5: Duncan’s test for expression data; Tables S6 and S7: Raw data of morphological characteristics of each group; Figure S1: Correlation analysis plot of morphological characteristics between all samples; Figure S2: Pairs plot between all samples; Figure S3: Pairs plot between control samples; Figure S4: Pairs plot between PEG-6000 treated samples; Figure S5: PCA plot containing replicate level averaged samples; Figure S6: PCA components weight distribution; Figure S7: Boxplot of maximum qunatum efficiency of PSII of Aksor and Gala varieties; Figures S8 and S9: Relative expression levels (fold) of drought-response genes faceted by days.
