# Supplementary material for: Gene Expression Analysis for Drought Tolerance in Early Stage of Potato Plant Development
Source: Biology (Basel). 2024 Oct 23;13(11):857. doi: 10.3390/biology13110857 (PMC11591961; doi:10.3390/biology13110857)
Supplement: Supplementary file 1 [file biology-13-00857-s001.zip › s2.pdf]

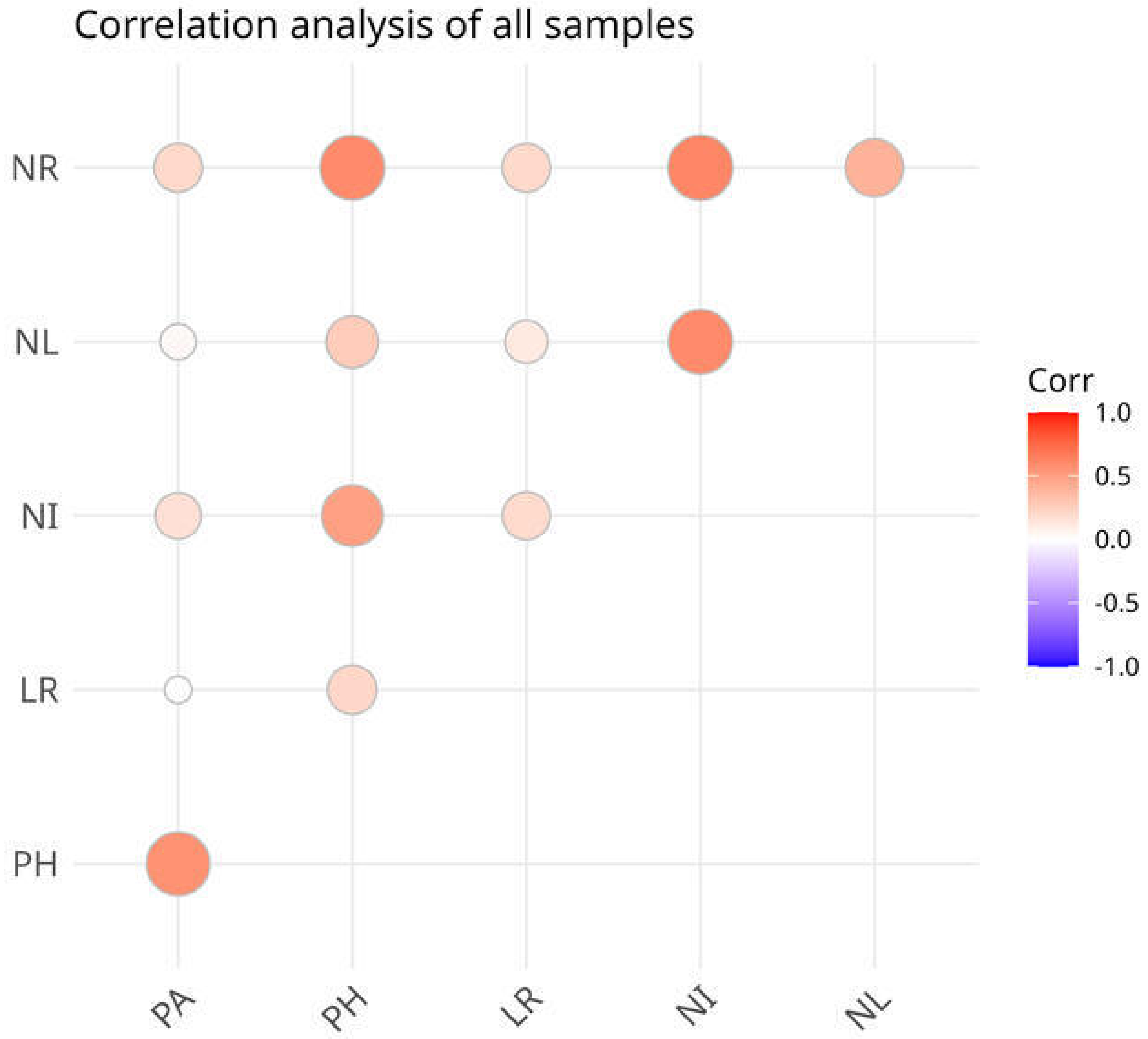
Correlation analysis of all samples
NR
NL
NI
LR
PH
PA
PH
LR
NI
NL
Corr
1.0
0.5
0.0
-0.5
-1.0

Pairs plot between all samples

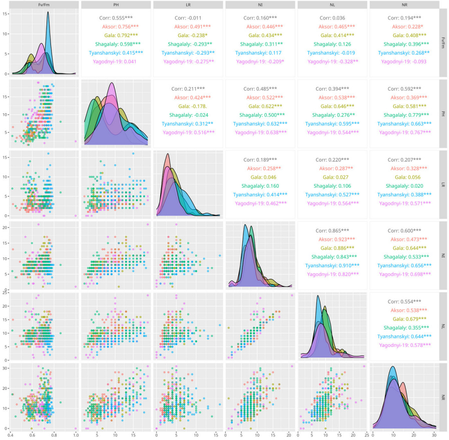

Pairs plot between control samples

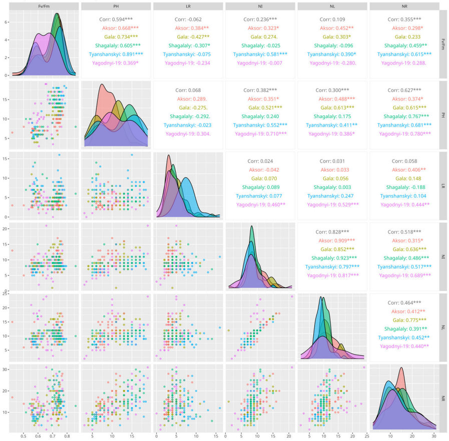

Pairs plot between PEG-6000 treated samples

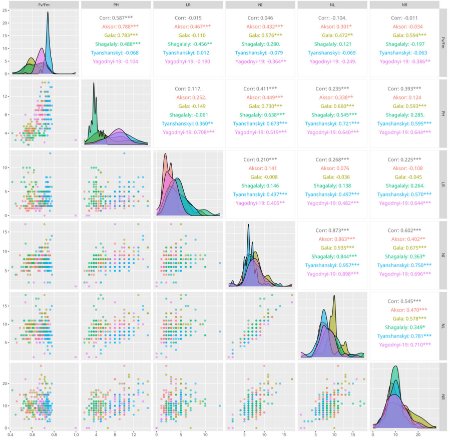

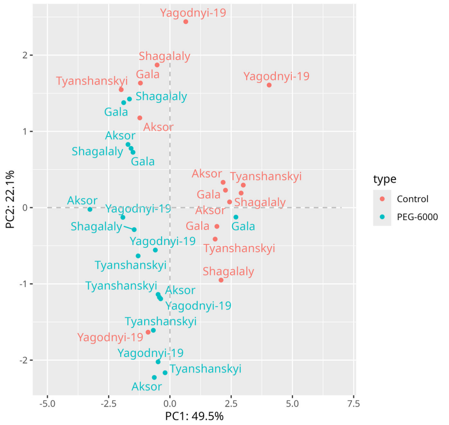

Yagodnyi-19
Shagalaly
Gala
Tyanshanskyi
Yagodnyi-19
Shagalaly
Gala
Aksor
Aksor
Shagalaly
Gala
Aksor
Tyanshanskyi
Gala
Shagalaly
Aksor
Aksor
Yagodnyi-19
Gala
Gala
Shagalaly
Yagodnyi-19
Tyanshanskyi
Tyanshanskyi
Shagalaly
Tyanshanskyi
Aksor
Yagodnyi-19
Tyanshanskyi
Yagodnyi-19
Yagodnyi-19
Tyanshanskyi
Aksor
PC1: 49.5%
PC2: 22.1%
type
Control
PEG-6000

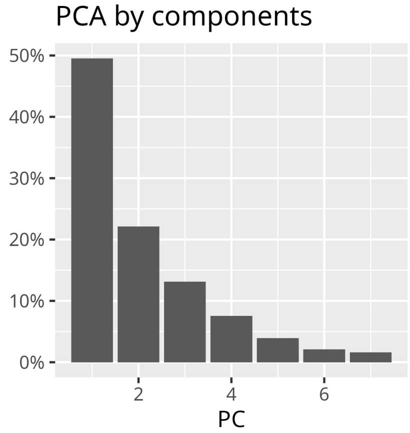

PCA by components
50%
40%
30%
20%
10%
0%
2
4
6
PC

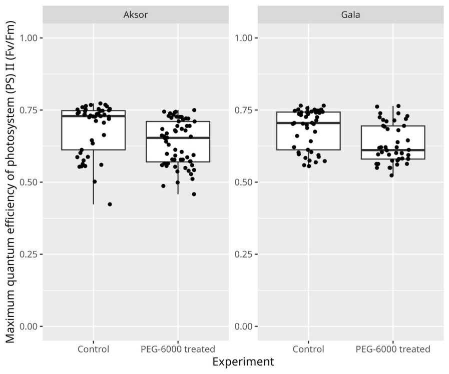
Aksor
Gala
1.00
0.75
0.50
0.25
0.00
Maximum quantum efficiency of photosystem (PS) II (Fv/Fm)
Control
PEG-6000 treated
Experiment

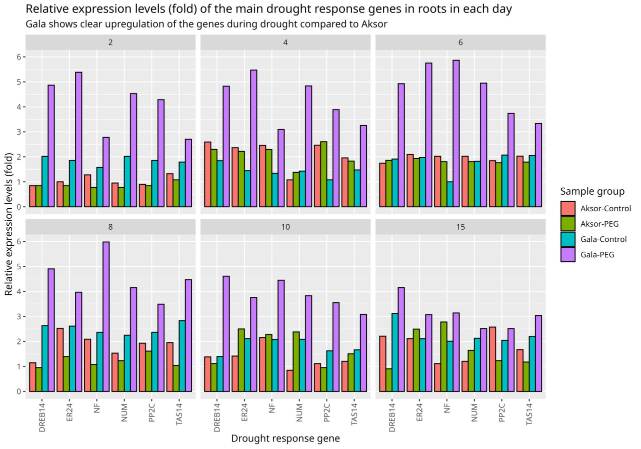

Relative expression levels (fold) of the main drought response genes in roots in each day
Gala shows clear upregulation of the genes during drought compared to Aksor
2
4
6
8
10
15
Relative expression levels (fold)
Drought response gene
DREB14
ER24
NF
NUM
PP2C
TAS14
Sample group
Aksor-Control
Aksor-PEG
Gala-Control
Gala-PEG

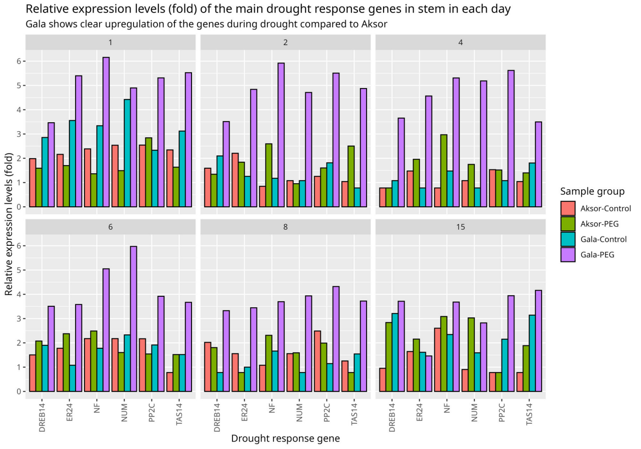

Relative expression levels (fold) of the main drought response genes in stem in each day
Gala shows clear upregulation of the genes during drought compared to Aksor
1
2
4
6
8
15
Relative expression levels (fold)
0
1
2
3
4
5
6
DREB14
ER24
NF
NUM
PP2C
TAS14
Drought response gene
Sample group
Aksor-Control
Aksor-PEG
Gala-Control
Gala-PEG
