# Supplementary material for: Gene Expression Analysis for Drought Tolerance in Early Stage of Potato Plant Development
Source: Biology (Basel). 2024 Oct 23;13(11):857. doi: 10.3390/biology13110857 (PMC11591961; doi:10.3390/biology13110857)
Supplement: Supplementary file 1 [file biology-13-00857-s001.zip › s3.pdf]

Gala (NORIKA GMBH, Germany)
Recommended region: North-Kazakhstan, Kostanay, Aktobe. Maturity date: Early maturing variety. Variety description: Plant of medium height, intermediate type, semi-upright. The leaf is large, intermediate, green. The waviness of the edge is weak to medium. Corolla medium-sized, white. Tuber is elongate-oval with small eyes. The rind is smooth to medium, yellow. Flesh is dark yellow. Yield: In Kostanay region average yield amounted to 24 t/ha. According to the results of production testing (Kostanay region) on the territory of farm "Terra" average yield amounted to 50 t/ha.

Yagodnyi-19 (Northwest Agricultural Research Center)
Medium-early variety, table purpose. The potato bush is tall and compact. The color of the tubers is white, the flesh is white and has a round-oval shape, as well as small and numerous eyes. Raw potatoes have a creamy flesh, which begins to darken slightly during cooking. The yield is 40-45 t/ha. The presence of starch 14 - 16 %. Flavor qualities are high. This variety of potatoes is resistant to viral diseases, can be multiplied by botanical seeds and leaf tubers. High stable productivity by years. It is raionized on the territory of Akmola, Aktobe and Karaganda regions.

Aksor ("Kazakh Research Institute of Potato and Vegetables Growing", LLP)
Origin: Created by intraspecific hybridization (Smachny x Olev) x Reserve with subsequent clonal selection. General characteristics: Medium-ripening table variety. The vegetation period is 80-100 days. Approbation features: The bush is tall, straight standing. Leaf coloring is light green, medium dissected. Flower corolla is medium-sized, white. The color of the rind is white. Flesh color is white. Tubers are roundish-oval in shape. Eye depth is medium. Yield: Potential yield 55 t/ha. Quality parameters: Tuber weight 110 g, starch content up to 22%. Possesses good storability and shelf life. Resistant to potato cancer. Recommendations: For cultivation in Aktobe, Almaty, East Kazakhstan, West Kazakhstan, Kyzylorda, Pavlodar regions.

Tyanshanskyi ("Kazakh Research Institute of Potato and Vegetables Growing", LLP)
Maturity: Early maturing variety. Vegetation period up to 100 days. Variety description: Half-spreading bush, tall, stems strongly branched, few, medium, leaves green, glossy, corolla white, tubers yellow, rounded, rough. Tuber flesh is yellow in color. Yield: The average yield amounted to 27.5 t/ha. Quality parameters: Starch content 17,4 %. Resistance to diseases: During the test diseases and pests were not damaged. Direction of use: Universal purpose. Suitable for industrial processing for chips and starch. Features: Tuber weight 97 g, marketability 91%, tasting score 5 points. Recommendations: Recommended for cultivation (under irrigation) in the mountain zone (Raiymbek GSU) of Almaty region.

Shagalaly ("Kazakh Research Institute of Potato and Vegetables Growing", LLP x A.I. Barayev Research and Production Center for Grain Farming)
In the conditions of Northern Kazakhstan on the material of potato varieties of domestic and foreign selection, parameters of the variety model for resistance to biotic and abiotic factors of the environment were improved. environmental factors. The number of days from mass sprouting to complete dying of the haulm variety Shagalaly 90 days, high-yielding, has field resistance to diseases and pests. Potential yield of 15.0-16.5 tons / ha. Starch content - 22,0 %. Weight of a marketable tuber 112,0-142,0 grams.
